# 133 Data-infrastructure Sport and Physical Activity in the Netherlands: studying data availability and quality for 164 indicators

**DOI:** 10.1093/eurpub/ckae114.011

**Published:** 2024-09-26

**Authors:** Suzanne van Mourik, Marjolein Duijvestijn, Wanda Wendel-Vos

**Affiliations:** National Institute for Public Health and The Environment (RIVM), Bilthoven, Netherlands; National Institute for Public Health and The Environment (RIVM), Bilthoven, Netherlands; National Institute for Public Health and The Environment (RIVM), Bilthoven, Netherlands

## Abstract

**Purpose:**

A lot of data is available on sports and physical activity in the Netherlands; however, a clear overview is lacking. Information can be challenging to find. This project aimed to provide an overview of available national representative data on indicators related to sports and physical activity, providing valuable information for policymakers, municipalities and researchers, and to foster harmonization in data collection and analysis by publishing source and methodology descriptions.

**Project description:**

In this project, the availability of nationally representative mean figures and/or prevalence rates was assessed for the 164 most relevant indicators for long-term monitoring in sports and physical activity (inventoried and divided over twelve topics earlier). If available, meta data on source and methodology descriptions for data collection and data handling were obtained from responsible researchers. Otherwise, it was investigated whether the indicator had been included in small-sample studies or in structural data collection, without publication of figures. Input was provided by 40 researchers, 3 policymakers, and 9 practice experts.

Webpages were developed for maintaining a dynamic, up-to-date overview and facilitating easy access to source and methodology descriptions.

To obtain the most comprehensive overview:

1. Essential components for the overview and source and methodology descriptions were established with research experts;

2. A preliminary inventory of the indicators was compiled by RIVM researchers;

3. Experts related to the topic of the indicators were approached individually and asked to provide input on the availability of mean figures and/or prevalence rates, and information about the source and methods used;

4. All inventoried information was evaluated by the experts per topic.

**Conclusions:**

The inventory showed that in January 2024 nationally representative information was structurally available for 74 (45%) of the indicators. Another 21 indicators (13%) could be provided with information based on existing structural data collection and 35 indicators (21%) had been previously studied in non-structural or non-representative data collections. For 34 indicators (21%) no information was available. The overview serves as a resource for policymakers, offering insights into available data and guiding investments in knowledge development. Furthermore, the overview facilitates researchers in sharing the quality of methodologies and enhances data comparability.

